# Effects of locally applied Insulin-like Growth Factor-I on osseointegration

**DOI:** 10.4317/medoral.22973

**Published:** 2019-08-19

**Authors:** Juan López-Quiles, Alba Forteza-López, Mónica Montiel, Celia Clemente, Jesús-Ángel Fernández-Tresguerres, Isabel Fernández-Tresguerres

**Affiliations:** 1 Associate Professor. MD, PhD, DDS. Department of Dental Clinical Specialities, School of Dentistry, Complutense University. Madrid, Spain; 2 Postgraduate student. DDS. Department of Dental Clinical Specialities, School of Dentistry, Complutense University. Madrid, Spain; 3 Private practice. DDS. PhD. Madrid, Spain; 4 Associate Professor. MD, PhD. Department of Human Anatomy and Embriology, School of Medicine. University of Alcalá, Alcalá de Henares, Madrid, Spain; 5 Full Professor. MD. PhD. Department of Physiology. School of Medicine. Complutense University. Madrid, Spain

## Abstract

**Background:**

The aim of this study was to assess the effect of local application of IGF-I on osseointegration of dental implants placed in osteoporotic bones.

**Material and Methods:**

16 rabbits were randomly distributed into two groups: eight animals were ovariectomized and fed a low-calcium diet for six weeks, in order to induce experimental osteoporosis, and the others were sham-operated and fed a standard diet. A titanium implant was inserted into the tibiae in both groups. In half of the rabbits, 4 μg of IGF-I was applied into the ostectomy, prior to the implant insertion. A total of 32 implants were placed. Animals were sacrificed two weeks after surgery and decalcified samples were processed for Bone-To-Implant Contact (BIC) and Bone Area Density (BAD) measurements. Analysis of variance (ANOVA) was used for statistical evaluation. *P*<0.05 was considered to be significant.

**Results:**

Ovariectomy induced statistically significant lower BAD values (*p*=0.008) and a tendency towards lower BIC values when compared osteoporotic and healthy groups. The administration of 4 μg of IGF-I did not produce statistically significant differences neither on BIC nor on BAD values, neither in the osteoporotic animals nor in healthy.

**Conclusions:**

Within the limitations of this experimental study, local administration of 4 μg of IGF-I was not able to induce any changes in the osseointegration process two weeks after surgery, neither in healthy rabbits nor in the osteoporotic group.

** Key words:**IGF-I, implants, osseointegration, osteoporosis, rabbits, BIC.

## Introduction

Bone is a dynamic tissue, that is continuously undergoing resorption by osteoclasts and formation of new bone by osteoblasts. Bone tissue is renewed 10% per year; the amount of renovated cortical tissue is 3-5%, while the reposition of cancellous bone is 25% per year. Thus, total skeleton is renovated completely after10 years (1). Implant placement can be considered as an aggression towards the bone tissue that undergoes a process of regeneration. An inflammatory response, hematoma, and a release of interleukins and growth factors constitute the cascade of events into the bone remodeling process (1). Among all factors, the growth hormone/insulin-like growth factor I (GH/IGF-I) axis is crucial for the regulation of bone formation. GH and IGF-I play an important role in the acquisition of bone mass during adolescence as well as in the maintenance of bone mass during adult stage. GH/IGF-I axis deficiencies lead to osteoporosis and bone-loss disorders (2). IGF-I is a small peptide, with a similar structure to insulin, that acts as a systemic and local regulator of skeletal growth. Circulating IGF-I is synthesized in the liver under GH control, and it is bound to IGF-binding protein-3 (IGFBP-3), among others (3). Circulating IGF-I mediates GH effects on longitudinal bone growth. Elsewhere, IGF-I is also synthesized locally by osteoblasts in response to parathyroid hormone (PTH), mediating its anabolic effects on bone (4). IGF-I and II are the most abundant growth factors stored into the bone extracellular matrix (5).

IGF-I synthesis by the liver, is regulated not only by GH (3), but also depends on the individual nutritional status. Thus, in anorexia nervosa, bone loss occurs, and it seems to be associated with a decrease in serum levels of IGF-I(6,7).

Osteoporosis is a skeletal systemic disease, characterized by a reduced bone mass, a deterioration in bone microarchitecture, and increased susceptibility for fractures (8). It is estimated that over 200 million individuals suffer from osteoporosis in the world, constituting a major public health problem (9). Although the relationship between skeletal and maxilla bone density has been controversial in the past, in the recent years it has been proven the influence of skeletal bone density on dental implant stability in patients with osteoporosis (10).

Animal studies have associated the presence of IGF-I and bone mass preservation. It has been proven that in knock out mice for IGF-I, osteopenia exists, confirming that IGF-I is an important factor in bone remodeling (11). In addition, it is known that circulating IGF-I contributes to cortical mass maintenance, while local IGF-I participates in the integrity of cancellous bone (12). Moreover, low IGF-I serum levels are also related to reduced bone mass in rats, which is recovered with the administration of low doses of IGF-I (13).

Human studies to evaluate the effects of IGF-I on bone turnover are limited. A decline in GH/IGF-I axis in the elderly may contribute to the pathogenesis of osteoporosis (14). In addition, it seems to exist a correlation between serum levels of IGF-I and bone mineral density (BMD) values in postmenopausal women (15), as well as reduced IGF-I levels are related with an increased risk for fractures (three times more frequently) (16), independently of other parameters such as BMD, age, duration of menopause, or body mass index (17).

Nowadays, therapeutic uses of IGF-I are limited to Laron Syndrome (severe primary IGF-I deficiency due to genetic GH resistance or insensitivity) (18,19). This indication was approved by the FDA (Food and Drug Administration) in 2005, and by the EMA (European Medicament Agency) in 2007. However, several clinical trials have been carried out in order to assess the possible efficacy of recombinant IGF-I in other pathologies such as diabetes I and II (20), ALS (amyotrophic lateral sclerosis), Alzheimer, anorexia nervosa or osteoporosis. In patients with anorexia nervosa, IGF-I administration induced an increase in BMD values and markers of bone turnover in women with severe osteopenia (7).

On the other hand, it is known that GH has a paracrine effect on bone, besides the well-known endocrine action (21). However, only a few papers have evaluated whether IGF-I could have a local effect on bone. It can be thought that if IGF-I is the GH mediator, IGF-I, locally applied, could have a beneficial effect on bone, like GH does (22). As IGF-I has not been yet used locally to improve the osseointegration process in osteoporotic rabbits, the objective of this study was to assess whether the local administration of IGF-I could enhance the osseointegration process in this osteoporotic animal model.

## Material and Methods

In the present study, a total of 16 six-month-old female New Zealand rabbits were used. This study was approved by the Local Committee of Ethics for the Use of Animals of the Complutense University of Madrid (UCM).

-Experimental osteoporosis 

A total of 16 rabbits were randomly distributed into two groups: a first group with eight animals, that were ovariectomized (ovx) and fed a low-calcium diet for 6 weeks, to induce experimental osteoporosis by the method previously described (OVX) (23). The rest of animals were sham-operated and fed a standard diet (HEALTHY).

-Implants insertion 

A total of 32 implants were placed into the rabbit tibiae. The implants were screw-type and manufactured from commercially pure titanium, 6 mm in length, and 3.3 mm in diameter, with an external hexagon (B&W®).

Under general anesthesia, titanium monocortical implants, were placed in the proximal part of the anterior aspect of the tibia, one centimeter below the anterior tibial tuberosity, in its middle face. One implant was placed in the left tibia and another one in the right tibia. In six of the rabbits (three HEALTHY and three OVX), implants were placed without another additional substance, and they were considered as controls (CONTROL/OVX and CONTROL/HEALTHY). In the other ten animals (five HEALTHY and five OVX) implants were placed with previous addition of 4 µg of IGF-I into the surgical site (GroPep. Thebarton. Australia) (IGF-I/HEALTHY and IGF-I/OVX).

In the preoperative period, the surgical field was shaved and disinfected with iodine povidone. The rabbits were anesthetized with Midazolam (Dormicum, Roche), Ketamine Hydrocloride (Imalgene 1000 Merial Laboratorio S.A. Barcelona) and Xylazine (Rompún 2% BAYER S.A. Barcelona). A full thickness flap was made with mucoperiosteal detachment and the implant site was prepared with the drills of the system at low speed (800 rpm) with saline solution irrigation. The implants were placed, and in the selected animals, 4 µg of IGF-I was applied before implants placement. Finally, the surgical wound was sutured by planes using 4/0 resorbable Vicryl suture. Postoperative antibiotic (oxytetracycline) and analgesic (buprenorfine) treatment was administered for five days.

-Histologic processing

Two weeks after implants placement, the animals were sacrificed with IV lethal sodium pentobarbital. Soft tissue was removed from the tibias and fixed in 10% formaldehyde in a buffered solution at pH 7. Samples were included in 2-hydroxyethylmethacrylate (Technovit 7200, Heraeus Kulzer, Germany) in growing concentrations for two months, following the method previously described (23). After polymerization, a homogeneous block of 20 mm was achieved. Transversal cuts were made by the Exakt cutting band microtome (Exakt Apparatebau. Norderstedt, Germany). Each section was grinded until a final thickness of 50-80 µm was obtained. The samples were stained by Masson´s, Toluidine Blue, Hematoxylin-Eosin, and were observed under light microcopy (Leica).

-Histomorphometric analysis

The morphometric study was performed by the MIP-45 imaging analyzer (Digital Image, Barcelona, Spain). Measurements of bone-to-implant contact (BIC) ± SD (standard deviation) were calculated. BIC was defined as the length of bone surface in direct contact with the implant perimeter x 100. BIC was measured following the method previously described (23).

Bone Area Density (BAD) analysis was also performed in order to measure the newly formed bone in the peri-implant area. It was calculated as the quotient of bone area between threads and the total area between threads multiplied in turn by 100.

All the measurements were made by a unique observer.

-Statistical analysis

For statistical analysis, SPSS 22.0 software was used. In order to assess the normality assumption of the sample, Shapiro Wilk test was applied. Paired test was applied to compare between the right and left tibiae data. Analysis of the variance (ANOVA test) for two factors was applied taking as the dependent variable the BIC or the BAD value, and as factors OVX and IGF-I. *p*<0.05 was considered to be significant.

## Results

-Histological analysis

In CONTROL/HEALTHY group, newly bone formation around the implant can be observed. Newly formed trabeculae from the periosteum and the endosteum at the cortical level can be seen. Neo-formed un-mineralized osteoid tissue at the medullar level can be distinguished.

In CONTROL/OVX group, lesser neo-forming activity than in the healthy group and greater presence of un-mineralized osteoid tissue at the compact bone cortex can be observed. Lesser presence of neo-formed trabeculae from the endosteum can be seen 

In IGF-I/HEALTHY group, histological characteristics similar to controls can be seen but with a less dense, shorter and thinner trabeculae than in the control group.

**Figure 1:**
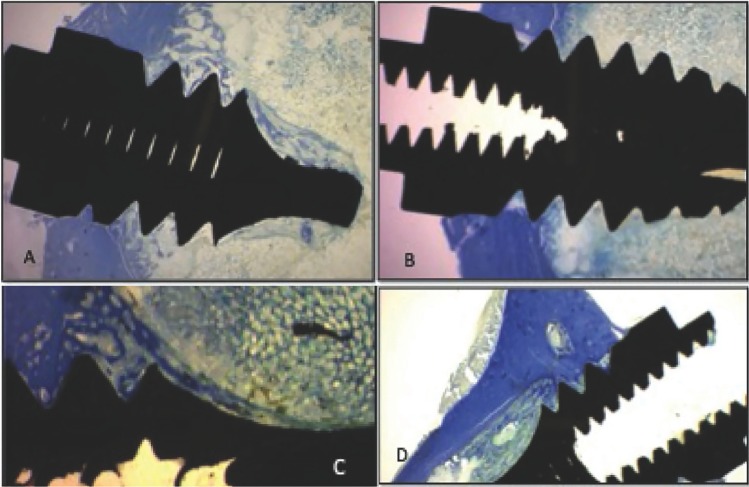
Comparison of the four study groups. Toluidine blue staining. A) CONTROL/HEALTHY group. B) CONTROL/OVX group. C) IGF-I/HEALTHY group. D) IGF-I/OVX group.

In IGF-I/OVX group, short and thin trabeculae can be observed. Limited bone activity is observed as well as slightly neo-formation of trabeculae and osteoid tissue is shown (Fig. 1) 

-Morphometrical Analysis

Bone to implant contact (BIC) and Bone Area Density (BAD) means ± SD were obtained by the MIP-45 imaging analyzer. The paired test showed no differences between left and right tibiae data, so all of them were considered for the statistical analysis. Shapiro Wilk test showed normality of the sample, so ANOVA test was performed.

Lower values of BIC in the OVX group respect to the HEALTHY group were observed, but this difference, was not statistically significant. However, in terms of BAD, statistically significant differences were found when OVX and HEALTHY groups were compared (*p*=0.008).

**Figure 2:**
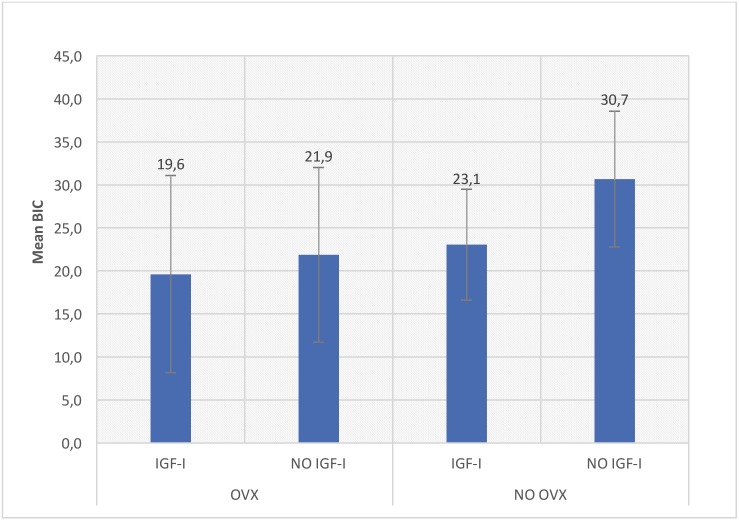
BIC values.

**Figure 3:**
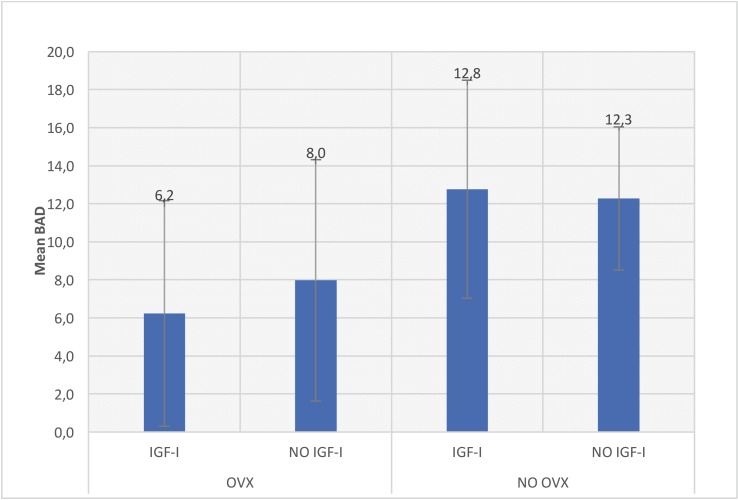
BAD values.

No significant differences were detected neither on the BIC nor on BAD with local IGF-I administration. Likewise, higher BIC values in both groups in the absence of growth factor were seen, although without statistically significance (Figs. 2,3).

## Discussion

Therapies against osteoporosis are antiresorptive or anabolic drugs. Bisphosphonates (BPs) are included into the first group, and teriparatide (1-34 PTH) belongs to the second one (9). However, in the recent years several novel anti-osteoporosis drugs have been developed, such as monoclonal antibodies against RANKL (Denosumab-Prolia®), or against sclerostin (a novel protein secreted by osteocytes which is a negative regulator of bone formation) (Blosozumab and Romosozumab, not allowed by FDA yet) (24). Their commercial availability is expected by 2020, since clinical trial are providing good results (25). Denosumab data indicate good results in increasing bone mass in osteoporotic patients, although adverse effects in the jaws have been registered, similarly than BPs (26). However, the prevalence of these side effects is minor with Denosumab than BPs, and they seem to revert when Denosumab withdrawal is carried out.

Due to current anti-osteoporotic drugs have been reported to have adverse effects, other anabolic substances such as GH and IGF-I have been studied for bone loss-related diseases treatment (9,27).

To our knowledge, this is the first study in which the effect of locally applied IGF-I on the osseointegration process in osteoporotic rabbits is evaluated.

The experimental osteoporosis model for the implant placement has been already validated by our group (28). This experimental animal model was obtained by a bilateral OVX and a low-calcium diet of 0.07% of calcium, instead of 0.14% in the standard diet, for 6 weeks. After this time a decrease in BMD of 4-10% was obtained depending on the studied area (calotte, tibia or cervical spine). This is the model the authors used in this current study.

-Osteoporosis and osseointegration

Several authors have studied the osseointegration characteristics in osteoporotic bone. The literature shows that induced osteoporosis in animals produces an alteration on the osseointegration process and a significant reduction in the BIC values. Thus, Lugero and colleagues (2000) (29) studied the osseointegration of two types of implants (threaded and cylindrical) in osteoporotic rabbits. The authors reported that bone formation is greater in the control group but, although osteoporosis affects the healing of bone tissue around the implant, prolonging the process, osseointegration can be achieved in osteoporotic bone. Duarte and collaborators (2008) (30) evaluated the influence of estrogen deficiency on the bone around implants in ovariectomized rats. The authors observed significant lower trabecular values in ovx rats versus controls.

However, despite considering osteoporosis as a possible contraindication for implants placement, there are several histological studies in humans in which implant osseointegration has been evaluated, obtaining high percentages of bone-to-implant surface that confirm the presence of osseointegration as well as the presence of healthy bone in intimate contact with implants surface (31,32), although the general recommendations are longer healing times in osteoporotic patients (10).

-IGF-I and osseointegration

Regarding the possible influence of IGF-I on the osseointegration process, animal experimentation has demonstrated the anabolic role of IGF-I on BMD, verifying a decrease in BMD if the IGF-I production or function is altered, and an increase in bone mass if exposure to IGF-I is increased (12,33).

However, clinical trials have shown that at low-doses, IGF-I increases bone formation, and at high doses, it increases bone remodeling in women with osteopenia due to anorexia nervosa (34). Although the function of IGF-I in the process of bone resorption is unclear, it is known that IGF-I induces the synthesis of RANKL and, subsequently, enhances osteoclastogenesis, increasing bone resorption (35). Thus, IGF-I activates the bone remodeling process as GH does (14).

A few studies combine different growth factors with IGF-I. Accordingly, Lynch and colleagues (1989) (36) were the first group to evaluate the osseointegration process after local administration of IGF-I+PDGF (Platelet Derived Growth Factor) prior to the implant insertion in dogs. At seven days, the percentage of implant surface in contact with the new bone was evaluated, being the data statistically significant.

The same combination of growth factors was used by Stefani *et al.* (2000) (37), who analyzed the osseointegration process at 3, 8 and 12 weeks. In their study, BIC values were proved to be greater at 3 weeks, rather than at 12 weeks, showing the beneficial effect of the combination of IGF-I+PDGF on the initial phase of the osseointegration process.

Similar results were demonstrated by Nociti and co-workers (2000) (38). In this study the application of PDGF in combination with IGF-I simultaneously with implants placement, showed a significantly higher BIC value and a greater percentage of bone area when compared with the controls.

IGF-I, osseointegration and osteoporosis

This is the first study which evaluates the influence of the local application of IGF-I on osseointegration in osteoporotic rabbits. In 2017, Xing and colleagues (39) published a study that evaluate the influence of IGF-I on titanium implants coated by layer-by-layer polyelectrolyte multilayers, under osteoporotic conditions. They concluded that the application of IGF-I could promote osseointegration in osteoporotic animals, since the local application of IGF-I seems to promote early adhesion of bone marrow mesenchymal stem cells as well as their differentiation. At eight weeks, the histological analysis showed greater bone-to-implant contact in test versus controls.

Our group was the first to demonstrate the effect of GH locally applied on the peri-implant bone reaction in an experimental animal model, both under osteoporotic conditions (22) and without osteoporosis (40,41), obtaining an improvement in peri-implant osteogenesis and higher BIC, 15 days after implant placement, with local GH treatment.

Considering that IGF-I is the GH mediator, it is conceivable that local administration of IGF-I could have a GH-like effect. However, in this current study, local administration of 4 µg of IGF-I did not induce any histological changes, neither on the BIC or BAD in OVX animals, nor in animals without osteoporosis, suggesting that the 4 µg dose may be very small compared to the 4 IU of GH (equivalent to 1.3 mg of GH). 

Regarding animals without osteoporosis, the application of IGF-I decreased BIC values, although without statistically significance. This could be due to the fact that IGF-I is able to accelerate the remodeling process (7) and, after 15 days, the resorptive phase could predominate over apposition. On the other hand, because the half-life of IGF-I is only three hours, it could be assumed that if its administration had been carried out by a continuous infusion pump or by encapsulation, which allow a sustained release, greater differences could have been obtained (42,43).

## Conclusions

In spite of the beneficial effects reported by other authors, and within the limitations of this experimental study, it can be concluded that local administration of 4 µg of IGF-I is not able to enhance the osseointegration process neither in the non-osteoporotic group nor in the osteoporosis animal model.
